# Fracture Analysis and Shear Strength of Aluminum/CFRP and GFRP Adhesive Joint in Fiber Metal Laminates

**DOI:** 10.3390/ma13010007

**Published:** 2019-12-18

**Authors:** Monika Ostapiuk, Jarosław Bieniaś

**Affiliations:** Department of Materials Engineering, Lublin University of Technology, Nadbystrzycka St. 36, 20-618 Lublin, Poland; j.bienias@pollub.pl

**Keywords:** fiber metal laminates, shear strength, glass and carbon fiber, failure

## Abstract

Fiber metal laminates (FMLs) were initially developed under the motivation of the aerospace industry. Generally, they consist of aluminum and high strength glass fiber in polymeric layers, but the new challenge is to apply them with a carbon fiber. Pretreatment of aluminum is the main factor responsible for the adhesion between metallic and polymeric layers. The shear strength test a very popular method in the experimental joint of two components. In this article, the main aim was to compare the surface pretreatment and configuration of fibers in FMLs based on aluminum with glass and carbon fibers. The decisive factor of strength in adhesive or cohesive failure is first the type of fibers, and second, the surface preparation.

## 1. Introduction

Fiber metal laminates (FMLs) consisting of metal sheets and fiber-reinforced composite layers were initially developed under the motivation of the aerospace industry in the middle of the last century [1]. The most widespread are laminates consisting of aluminum and high strength glass fiber that are currently the main type of reinforcement used in laminates [2]. Glass fiber is characterized by low density, high fatigue strength, excellent static properties and high impact resistance, and by satisfactory corrosion resistance [3,4,5,6]. Due to the continued growing interest in hybrid laminates, the new challenge is to use titanium, magnesium, or aluminum–lithium alloys. The laminates containing carbon fiber seem to be a favorable solution. It can be expected that such laminates are characterized by high stiffness with good impact properties and great strength, constituting an advantage, especially for applications in space [1]. Low density and weight saving materials seem to be attractive thanks to the phenomena of bonding two completely different materials and the surface preparation. However, in this case, it is necessary to choose the correct surface pretreatment in order to obtain high strength properties at the interface between the polymer and metal layers. Generally known mechanical and electrochemical processes (i.e., phosphoric acid anodizing (PAA), chromic acid anodizing (CAA), and sulfuric acid anodizing (SAA) [1,7]) are the most popular methods of surface preparation to achieve the desired results associated with the interface metal—polymer composite joint. Theoretically, the configuration of the joint, the thickness and local geometry of the adhesive and adherends, surface preparation, and environment together have significant effects on the failure of the joints. However, some parameters like the materials, thickness of adherends, and overlap length are usually restricted in practical applications. Based on the existing constraints, the effective optimization of joint design to achieve high load transfer efficiency is of great interest. A review of the article written by Shang et al. [8] showed those techniques applied to improve the strength of adhesive joints with composite adherends numerically, and an experimental study was conducted to investigate the influence of four manufacturing parameters (adherend thickness, bond-line thickness, bonding process, and surface preparation) on the shear behavior of (Glass Fiber Reinforced Polymer) GFRP-to-aluminum single-lap joints under static tensile loading. Analysis of the manufacturing parameters on the shear strength of aluminum/GFRP co-cured and adhesively bonded single-lap joints was presented by Çalik [9], who investigated the strength of different end parts of the adherend. Budhe et al. [10] generally discussed the effects of geometry and material property on the strength of adhesive joints. The impact of the thickness of the adhesive bonded layer on bonding forces in single-lap joints (SLJs) was also tested by da Silva et al. [11]. Sekercioglu et al. [12] found that the strength of the adhesive was affected by various factors including material properties, temperature, and surface roughness. Moreover, Melcher and Banea pointed out that temperature also affects the fracture behavior of adhesive joints [13,14,15]. Alfano et al. [16] indicated that surface pretreatment was required for reliable adhesive bonding. For this reason, they investigated the effect of a pretreatment method on the strength of Al/epoxy joints. In refs. [17,18], the authors discussed the effect of various damage and defects, and found that this one of the most important factors in the analysis of adhesive joints. Moreover, the development of cracks or debonding in the composite materials as an adherend also has an effect on stress distribution in the adhesive layer. Botelho et al. [19] and Park et al. [20] concluded that in Glare^®^ laminates, the critical challenge is to find practical methods of surface treatment in order to obtain a durable structure because the interface bonding the aluminum and epoxy–glass layers plays an important role in load transfers through the structures. Botelho [21] mentioned that it was possible to manufacture hybrid laminates with proper consolidation between the polymer composite and metal layers thanks to good wettability, because there is no visible presence of voids (porosity) in the metal layer at the interface with the composite [21]. On the basis of tests of composites reinforced with carbon fiber joints, Liu et al. [22] demonstrated that delaminations generated by shear strain were the basic form of damage in adhesive bonded structures. It was demonstrated that adhesive properties are affected by the molecular structure of the polymer, chemical properties, and the roughness of the metal substrate surface. They carried out peel tests indicating that the orientation of the metal sheet’s rolling direction and chemical surface properties caused by various changes in humidity in the environment had a significant impact on the adhesion [23,24]. Kellerman [25] and Ochoa [26] described certain factors that promoted the occurrence of adhesion including wettability and surface energy parameters. They outlined that the said factors were correlated with the high strength of bonding that was measured in shear strength tests. In a single shear test, there was loss of cohesion in two different areas, and adhesive loss of cohesion took place in the area of damage that occurred in contact with the steel surface. Cohesion means a stronger bonding on the interface (e.g., shear strength between layers and delamination resistance). A different observation was carried out by Uehara and Sakurai [27], who tested specimens made of carbon steel and found that, in the case of joints with epoxy resin, surface roughness did not lead to any significant changes in adhesion force in a single shear test.

However, the complete understanding of and control over the chemical and mechanical interactions in the scope of the adhesion mechanism have not been achieved yet. The factor preventing the complete understanding of adhesion mechanisms and the forecasting of the lifetime is associated with the fact that adhesion areas mainly affecting the adhesion and lifetime are situated at the interface point (i.e., in the oxide area and physical surface of the polymer). Such areas are not easily accessible for chemical or morphological analysis.

This article deals with the shear strength test behavior of CFRP/aluminum and GFRP/aluminum laminates and analyzed the morphology of failure on the interface between composite and metal. In fact, the aim of this research activity was the evaluation of the influence of surface pretreatment of aluminum on SLJ’s strength. Moreover, in this work, the glass and carbon fiber reinforcement of the composite material is shown in different configurations to explore the influence of the mechanism of the failure in SLJ tests in connection with surface morphology.

## 2. Materials and Methods 

### 2.1. Materials

The subject matter of the tests encompassed fiber metal laminates in a 2/1 configuration based on unidirectional glass/epoxy prepreg tape (nominal thickness: 0.25 mm, Hexcel, Stanford, CT, USA) and carbon/epoxy prepreg tape (nominal thickness: 0.131 mm, Hexcel, USA). The nominal volume fraction of reinforcing fibers was equal to about 60%.

Individual laminate components consisted of metal layers made of 2024 T3 aluminum alloy 0.5 mm thick. Two types of surface preparation were applied in the anodizing process in an aqueous solution of chromic acid (CAA) and sulfuric acid (SAA) and the surfaces of the anodized metal sheets were coated with a primer—an agent activating the surfaces—based on a polymer resin (3M Scotch-Weld Structural Adhesive Primer EC-3924 B).

**Figure 1:**
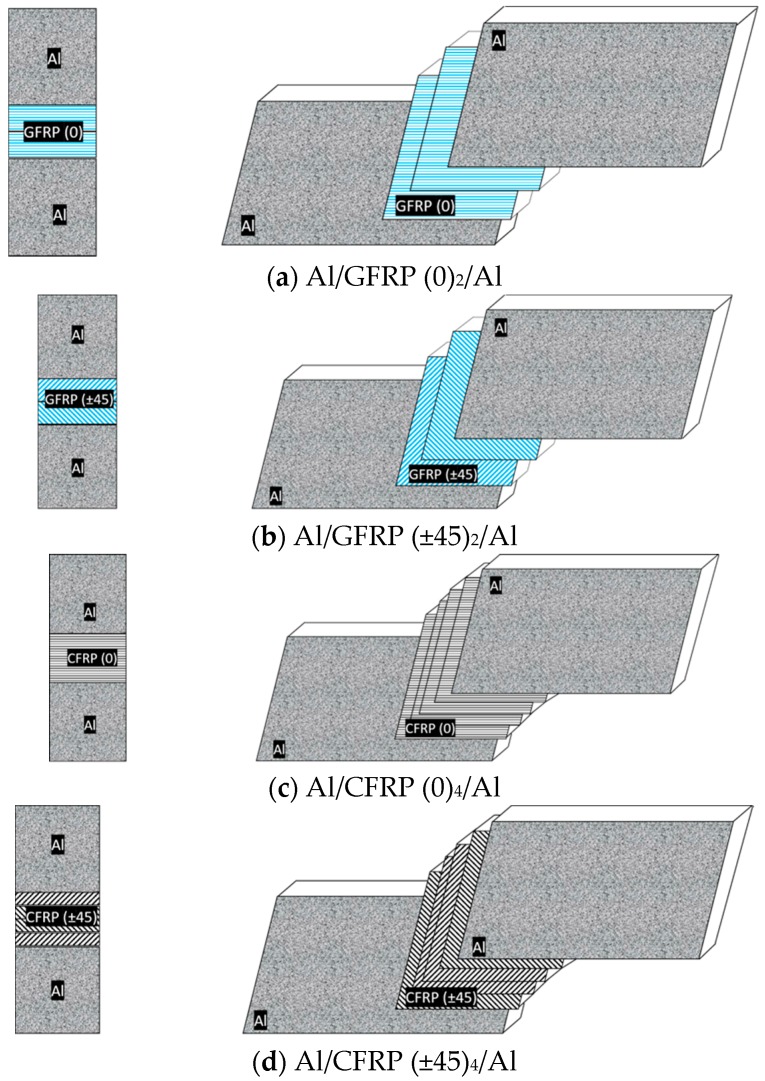
View of the fiber metal-laminates (FML) configurations.

The composite panels were fabricated in an autoclave process (pressure of 0.45 MPa, under pressure of −0.08 MPa, curing temperature of 135 °C, curing time of 2 h, heating/cooling 0.033 K/s). The laminates (Figure 1) were in the following configurations for CAA+P and SAA+P. The 2 and 4 represent the number of prepreg layers in one FML configuration.

### 2.2. Surface Measurements

The physicochemical adhesive properties of the surface layer were determined by means of wettability testing using measurements of wetting angle. The direct angle measurement was carried out for fluid drop (distilled water, diiodomethane)/surface. The value of surface free energy (SFE) was determined by means of the Owens–Wendt method calculated on the basis of the wetting measurements of the examined surfaces. The whole analysis for determining the surface free energy uncertainty by means of the Owens–Wendt method (also known as the Owens–Wendt method) was shown by Rudawska [28,29].

The measurements were carried out on aluminum surface specimens without previous surface preparation and after anodizing in CAA and SAA, and using the primer on both types of anodized surfaces.

In order to determine the basic geometrical parameters of the surface layer in materials being tested and surface topography (FRT Micro’Prof), scanning profilometers were used. The isometric view was completed for aluminum specimens without surface preparation and after anodizing in CAA and SAA and using the primer on both types of anodized surfaces.

### 2.3. Shear Strength Test

**Figure 2:**
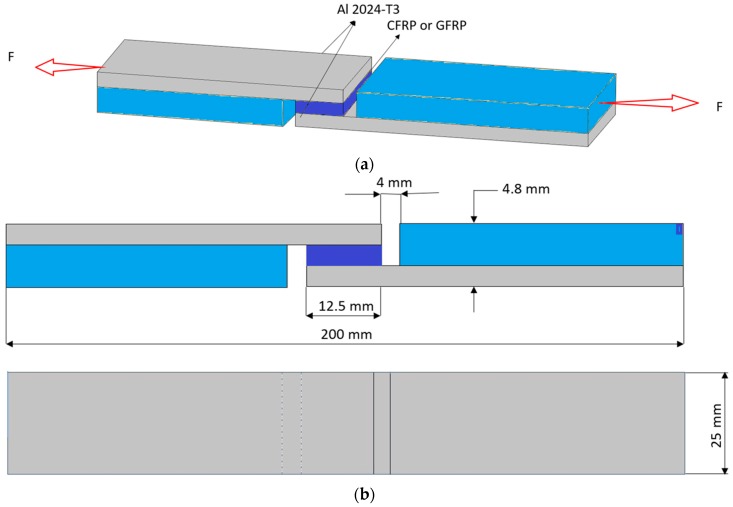
Scheme of single lap shear specimen test configuration: (**a**) schematic view and (**b**) profile with dimensions.

The single lap joint test tests were carried out in accordance with the ASTM D 3165–00 standard [30] on a Zwick Z150SN universal testing machine with test speed of 2 mm/min. The schematic sample view and profile with dimensions are shown in Figure 2.

Equation for shear strength (R_s_) destruction lap joint loaded:

(1) $$Rs\frac{F}{As}$$

where *F* = force (N) and *A_s_* = surface area (m^2^).

### 2.4. Microstructural Analysis

High-magnification for fracture surface observations utilizing ZEISS ULTRA PLUS, Germany) scanning electron microscopes were carried out using secondary electron (SE) imaging under 3.0 and 30.00 kV accelerating voltage conditions. Low vacuum pressure with 100 Pa was used to obtain a high quality of the fracture details.

## 3. Results

### 3.1. Surface Analysis

The results of the tests presented below encompass the analysis of the 2024-T3 aluminum alloy surface after the CAA and SAA process. 

Table 1 presents the results of the wetting angle and surface free energy (SFE) measurements that have been carried out by means of water and diiodomethane.

In the range of 43.9 mJ/m^2^ to 72.2 mJ/m^2^, the measurement of surface free energy for materials subjected to tests are included. On the basis of comparison with metal sheets without previous surface preparation, the value of surface free energy increased in metal sheets after anodizing. It was recorded that there was no surface preparation and the recorded value was equal to 43.9 mJ/m^2^. The highest value of surface free energy (by 64.5%) was observed in the case of the aluminum alloy anodized in CAA. Anodizing in SAA was the average value between CAA and no surface preparation; it was equal to 66.49 mJ/m^2^.

The wetting angle demonstrated that the angles for both fluids were less than 90° and are classified in the hydrophilic category. The values of the wetting angle were reduced after surface anodizing, particularly in the case of the aluminum alloy in CAA by about 75% for water and 65% for diiodomethane. In case of SAA, the value of the wetting angle was reduced by about 60% for water (i.e., it was equal to about 40%) and by 17% for diiodomethane (i.e., it was equal to about 83%) of the values recorded for metal sheets without previous surface preparation. The results of the tests carried out after previous surface preparation in the form of anodizing in CAA indicated the lowest values in the case of the wetting angle measurements simultaneously with the highest value of surface free energy. Consequently, it can be concluded that the obtained surface layer on metal is characterized by excellent adhesive properties.

**Figure 3:**
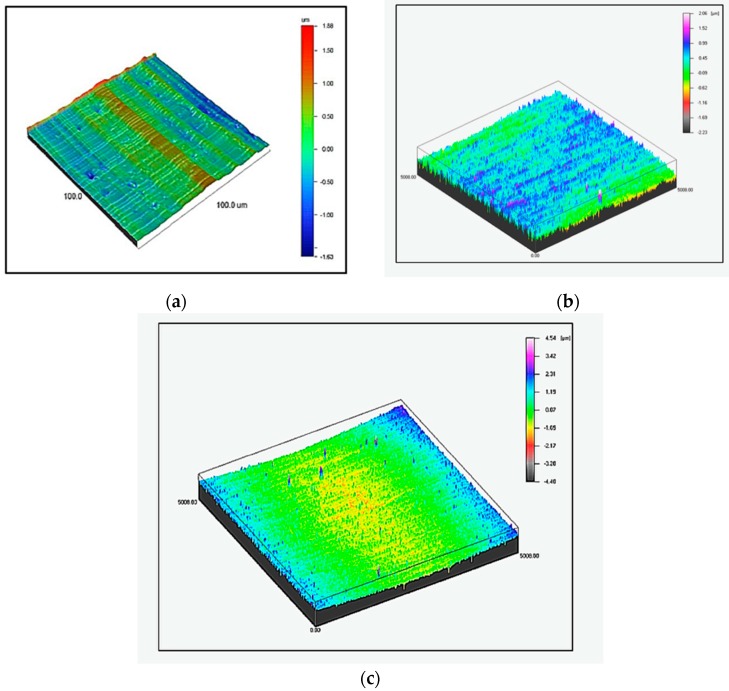
Isometric view of the aluminum surface: (**a**) without anodizing, (**b**) chromic acid (CAA) and (**c**) sulfuric acid (SAA); profilometer 3D.

However, the parameters after SAA showed values included in the range between the values obtained for the metal sheet without previous surface preparation and the values obtained after anodizing in CAA. Due to the fact that withdrawing the processes based on hexavalent chromium compounds is recommended, positive results in the scope of the wetting angle and surface free energy obtained for anodizing process in SAA gave us hope that it will be possible to substitute the CAA solution with the SAA solution. Figure 3, illustrating the representative examples of isometric images of tested specimens, constituted the graphical representation of roughness parameters. From the comparison of the isometric views obtained, differences were found in surface geometry depending on the substrate preparation method.

In the course of the analysis of the surface isometric view (Figure 3), it has been observed that as a result of different methods of surface preparation, the surface geometry obtained was different from the aluminum surface structure before anodizing (Figure 3a).

In the case of metal sheets without anodizing, it is possible to simultaneously observe rare indentations and irregularities with visible surface waviness. The surface of the aluminum CAA (Figure 3b) layer is characterized by a significantly higher number of irregularities with a high degree of distribution uniformity on the surface than without anodizing and after SAA. The surface of aluminum after SAA was characterized by a uniform structure (Figure 3c). Only in the case of aluminum CAA was a visible “pattern” observed on the surface.

On the basis of the test results presented in Table 2, it was found that the values obtained by characterizing the surface roughness were affected by the preparation process of the aluminum surface. In the case of the aluminum CAA and SAA layer application, the results obtained for the surface roughness parameter R_a_ were similar (a difference of 0.02 µm).

**Figure 4:**
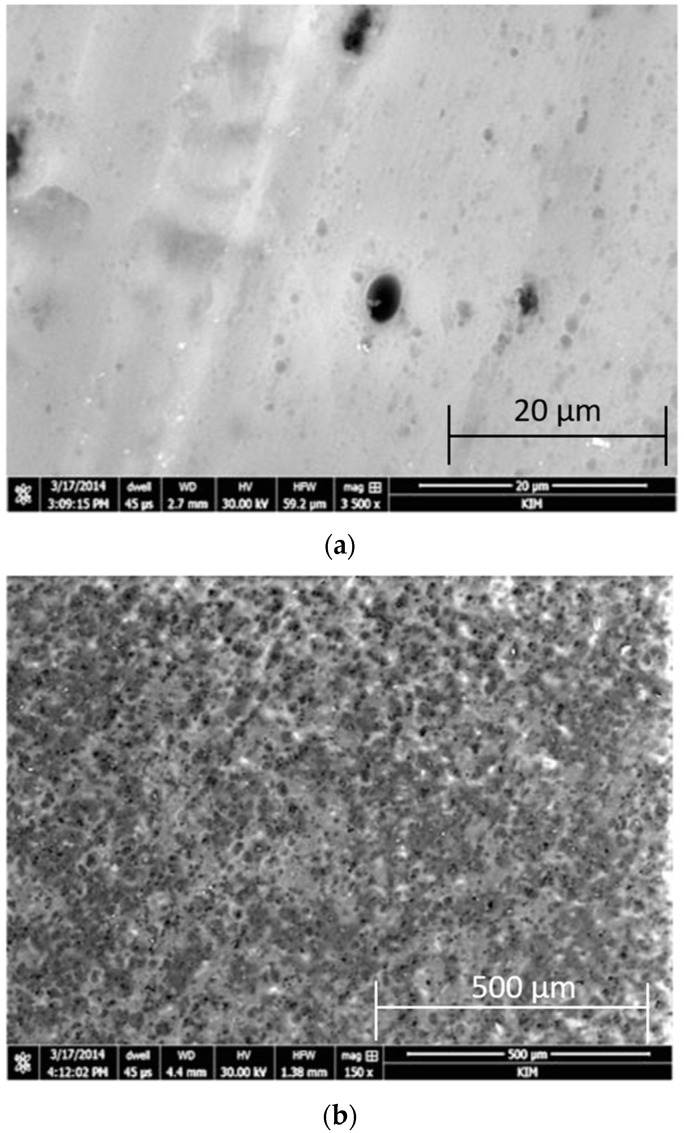
Surface morphology of: (**a**) CAA and (**b**) SAA; SEM.

Surface morphology is one of the factors that could be responsible for the strength in the joint bonding. Figure 4 presents the scanning electron. Scanning electron microscopy (SEM) images of layers anodized in CAA and SAA.

The observations of the surface morphology on the anodized layers indicate the satisfactory quality of the created oxide coats. A specific porous structure is visible, but in the case of anodizing in SAA (Figure 4b), this structure is significantly finer. It is also possible to observe the contours of pores in the form of darker spots after the anodizing process by filling them with a visible sealing layer. Aluminum with a primer after anodizing in CAA (Figure 4a) was characterized by a clearly smoother surface, but with locally occurring pores larger than those in the case of anodizing in SAA.

### 3.2. Shear Strength Test

Shear strength tests for the aluminum–epoxy and glass composite were carried out for two fiber configurations in the direction 0° and ±45° for anodizing in CAA and SAA.

From the analysis of the test results with respect to the values of the average breaking stresses (Table 3), the highest values were obtained for specimens with a fiber configuration at an angle of ±45°. Simultaneously, the highest value equal to 5.69 MPa for breaking stresses occurred for aluminum (SAA+P) and the lowest values were obtained for the same anodizing process, but in a fiber configuration at an angle of 0°. The values of breaking stresses of 3.13 MPa for a fiber configuration at an angle of 0° and (SAA+P) were the lowest ones from among the configurations subjected to tests. It was possible to observe the highest force required for the joint destruction for a fiber configuration at an angle of ±45° in both anodizing cases for carbon fiber. Any influence of the kind of fibers on a single lap joint test was not observed.

### 3.3. Fractography Analysis

**Figure 5:**
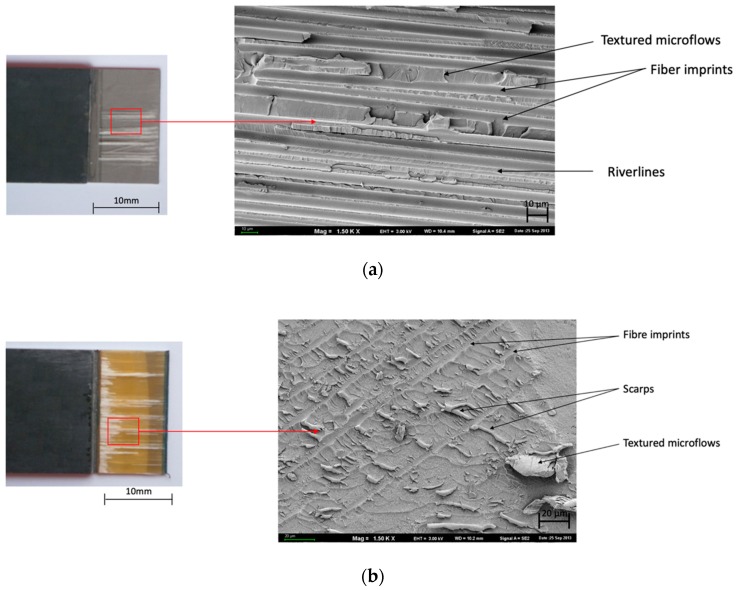

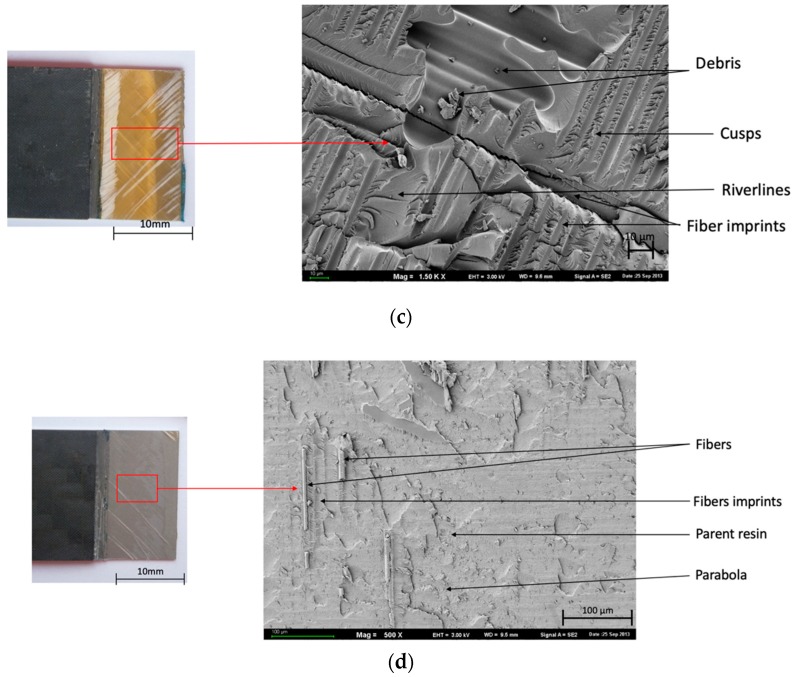
Surface morphology of CAA+P after the test. Configuration of the glass fibers: (**a**,**b**) 0° and (**c**,**d**) ±45°; SEM.

Figure 5 illustrates the macroscopic and SEM surface after the tests with both sides of failure. The comments on the surface morphology for both glass and carbon epoxy layers are included in the figures below.

Figure 5 confirms the adhesive-cohesive nature of the joint and the concentrations of glass fiber occurring on the specimen, and disclose “fiber imprints” (Figure 5b) on the destruction surface. The occurrence of “cusps”, which are specific for shear force impacts and the effects of resin “textured microflows” and “debris” (Figure 5b) were also observed.

**Figure 6:**
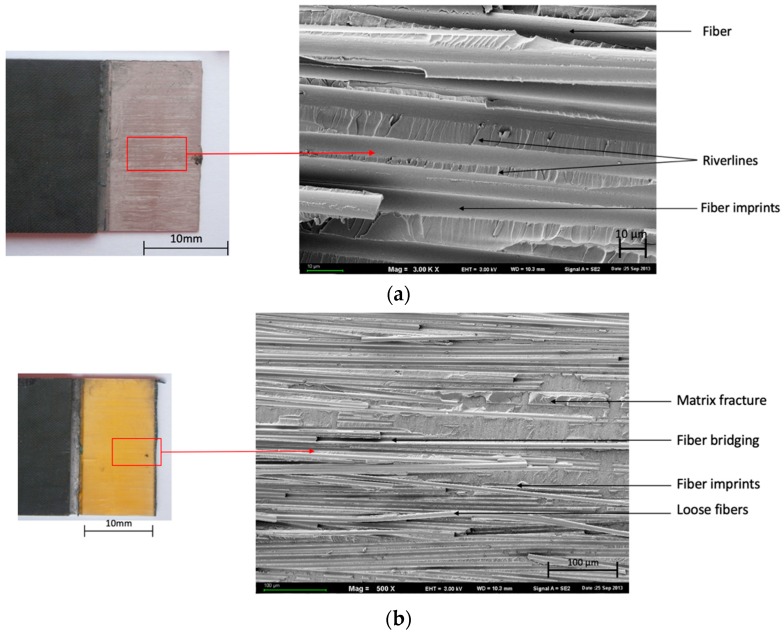

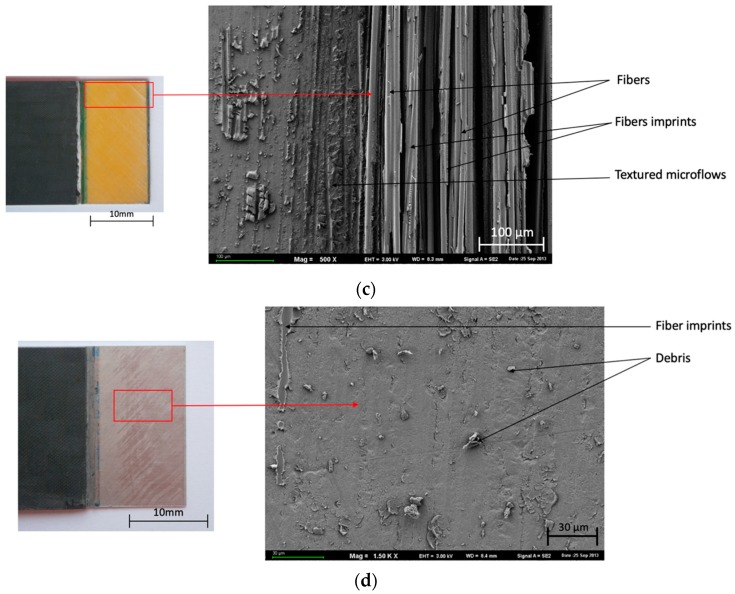
Surface morphology of SAA+P after the test. Configuration of the glass fibers: (**a**,**b**) 0°, (**c**,**d**) ±45°; SEM.

In Figure 6, the adhesive-cohesive nature of the joint “fiber imprints” and “river lines” (Figure 5a) are visible on the destruction surface. “Debris” and “fiber imprints” (Figure 6a) were observed.

**Figure 7:**
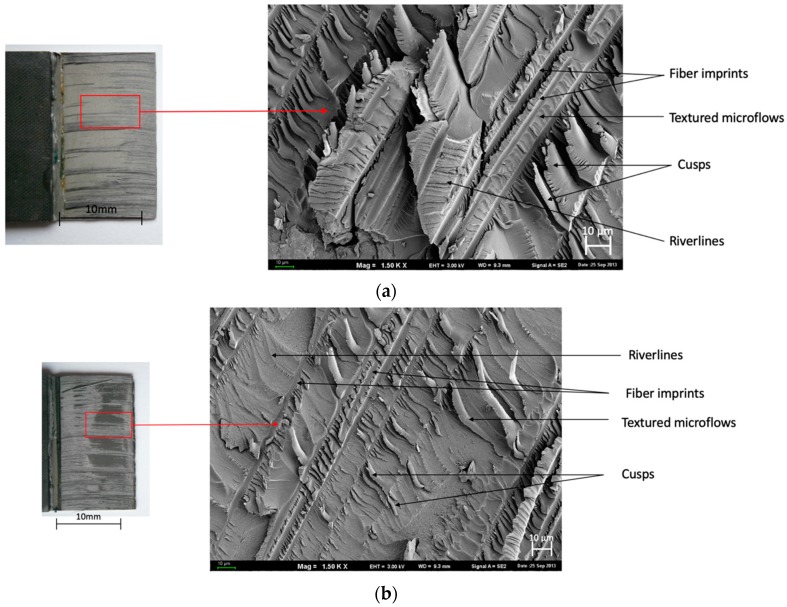

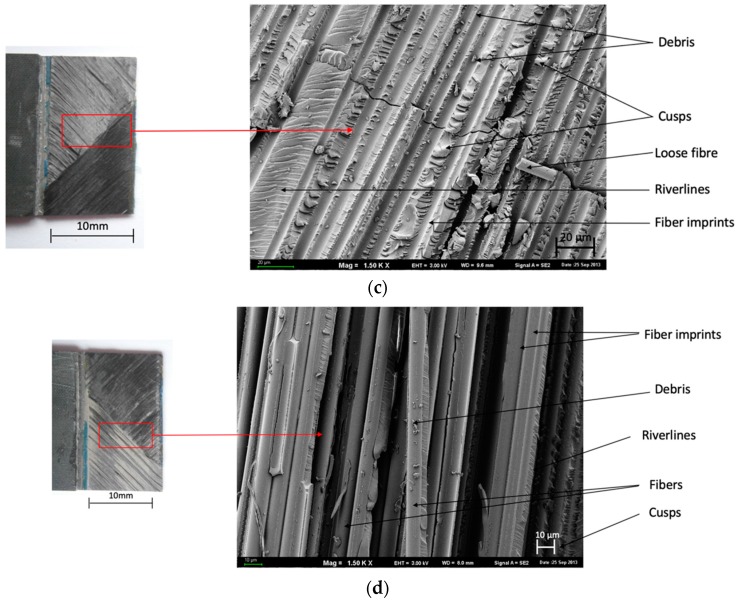
Surface morphology of CAA+P after the test. Configuration of carbon fibers: (**a**,**b**) 0° and (**c**,**d**) ±45°; SEM.

On the surface morphology in Figure 7, many characteristic degradations with carbon fibers were observed. Both the 0° and ±45° configurations showed “fiber imprints”, “river lines”, and “cusps”.

Table 4 presents the dominating characteristic shear failure in a combination of FMLs. Adhesive-cohesive (ADH+COH) failure is dominant for glass fiber independently of the surface pretreatment for carbon fiber only in (SAA+P) and [0°].

**Figure 8:**
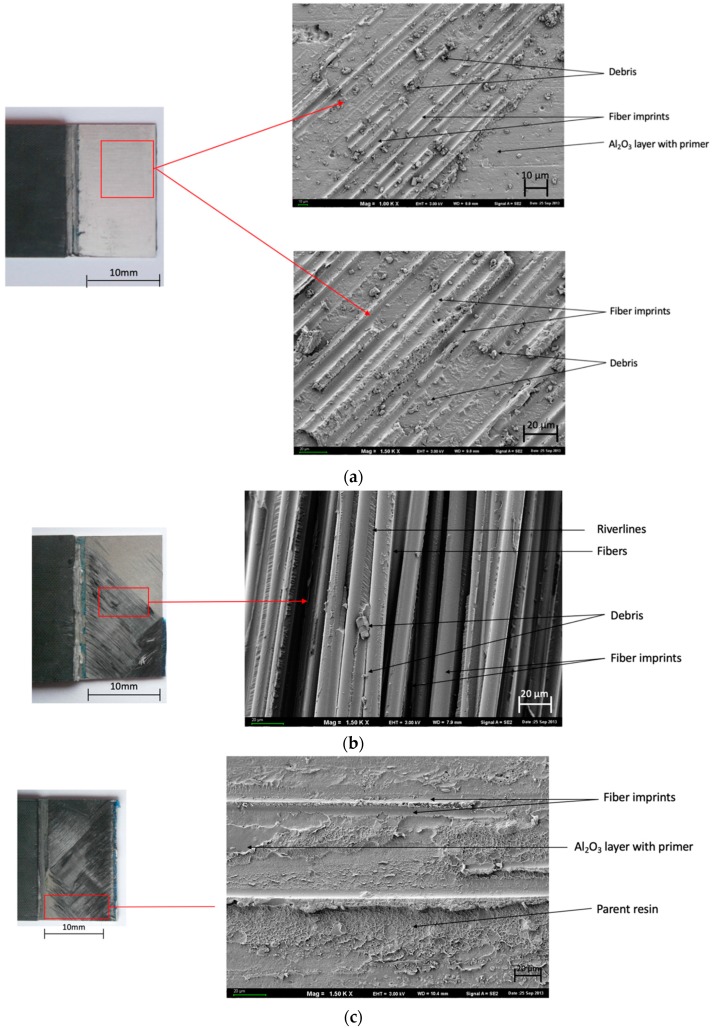
Surface morphology of SAA+P after the test. Configurations of the carbon fibers: (**a**,**b**) 0° and (**c**,**d**) ±45°; SEM.

In the course of the experiments carried out, it was observed that the manner of joint degradation could point to the type of anodized coat, which was disclosed for layers with a configuration of ±45°, characterized by the limited degree of matrix fiber fractures in the case of glass fiber (Figure 5b and Figure 6c). Significant degradation was observed in the case of carbon fiber characterized by clearly visible fractures in the middle part of the overlap (Figure 7b and Figure 8c).

The joints with metal anodized (CAA+P) were characteristic elements until the loss of continuity occurred at a lower breaking stress (Figure 5 and Figure 7). However, in the case of SAA+P (Figure 6 and Figure 8), the joints bear higher shear stresses with significantly lower displacement effects of the components. Both types of specimens are characterized by adhesion-cohesion (ADH+COH) occurring in composites through the delamination of resin in the vicinity of the metal-composite boundary. The polymer on the SAA+P is characterized by brittle—plastic fractures correlated with lower displacement in the course of shear force action (Figure 6 and Figure 8). In the case of laminates with an orientation of 0°, the shearing process also takes place with adhesion (ADH) occurring in the composite but with a higher intensity of fiber fractures (Figure 7). The layer characterized by adhesive bonding with the substrate is also characterized by a higher content of fiber fragments and by brittle—plastic fracture features in the matrix in the vicinity of the boundary with the layer anodized in sulfuric acid. The destruction morphology for the polymer composite and the surface of the metal sheet makes it possible to identify the growth of the material destruction. The orientation of concentration points for “river lines” may indicate the direction of the fracture growth.

The shear strength tests for the joints were selected to evaluate the impact of the laminate structure on mechanical properties that can be the measure of joint effectiveness. In the course of the single lap joint test, the specific inclination in the overlapping area (bending effect) until the time of destruction in the overlapping area was observed in each specimen. This phenomenon can be explained by the deviation of the direction of stresses occurring in the polymer composite layer. The theory of shear delay indicates that shear stress distribution is highly nonlinear in the force direction. Horizontal stresses occurring on superimposing edges, particularly in the middle of the joint line, are characterized by low values. The different rigidity and thickness of the materials contribute to a combination of shear and tensile forces [31].

In these tests, using joints that were subjected to shear forces, the layer of polymer composite in a fiber configuration of ±45° was characterized by the highest values of breaking stresses. The highest value of breaking stress occurred in SAA+P, but the lowest value was obtained for the same anodizing process in a fiber configuration of 0°. In Massuda et al. [32], they indicated a relationship between the value of roughness Ra and maximum breaking force F. In their opinion, the increase in the Ra parameter is accompanied by a reduction of the breaking force. Simultaneously, in the case of CAA+P, they obtained the opposite effect. In connection with the foregoing, the tests carried out in the present study did not exactly confirm the relationship between the roughness and maximum breaking stress: the isometric views were different for chromic and sulfuric acid, but the values of the shear stress did not exactly correspond to the surface preparation. First of all, the values of the breaking stress were conditioned by the fiber configuration in the polymer composite. Furthermore, the macroscopic observation in the course of shear process and after the test as well as the microscopic tests could indicate the impact of the anodized layer type on the shear mechanism.

The surface morphology of the metal sheets after the tests indicated the differences depending on the fibers’ layout in the laminate, which are the results of the observations of the damage mechanisms in the present study and are consistent with those presented by Greenhalgh [33], but were only associated with polymer composites. For specified layouts of the fibers and their degradation states under the impact tensile forces, the determined specific features of damage morphology precisely correlated with the test results. The mechanism of laminate destruction indicated the cohesive failure in the matrix around the fibers and between them as well as at the matrix–fiber interface [34]. In the case of a low strength of the matrix-fiber, the damage mechanism occurs in the matrix after the fiber boundaries [34,35]. Moreover, the substrate surface treatments and the direction of the fiber configuration may have a significant impact on the strength, in particular, in the case of joints exposed to environmental impacts. In principle, the stresses occurring in the joint are not uniformly distributed along the joint line. Therefore, the bonding force depends on the adhesive properties and joint structure. Consequently, the metal surface preparation belongs to the important aspects responsible for the creation of a reliable joint [21]. As a lower wetting angle means better wettability, it has been found that CAA is a better method of surface preparation for applications associated with the fabrication of FMLs as concluded by Critchlow et al. [36]. However, it does not exactly prove the results in this article where SAA could be an alternative to CAA as found in the results. It follows from the foregoing that surface roughness and stable oxide layers belong to important parameters contributing to a strong and durable joining of two materials into an integral structure that has a similar point. The advantages resulting from surface roughness encompass the increase in the specific surface as a result of introducing the morphology [37,38]. This factor can, to a certain degree, explain the increased welding efficiency on uneven surfaces. A lower surface roughness promotes a smaller angle of contact, better wettability, and higher joint strengths, which was proven in this research and is in accordance with Kwang-Soo, Pereira, and Tzetzis [39,40,41]. The increase of wetting angle vs. surface roughness increases the capacity of bonding and load transfer on the interphase boundary [30,39,40,41,42].

Generally, the opinions and research are divided, but the adhesion of the surface is extremely important because the presence of a weak inner interphase boundary may lead to a premature failure in the oxide, brittle hydroxide, or between the oxide and the hydroxide in the metal substrate. There are reports that the strength of the joint in Glare^®^ laminates is negatively affected by the occurrence of porosity in the interphase boundary, and the occurrence of various fracture mechanisms on the interphase boundary [30]. FMLs are able to absorb significant amounts of energy through localized fiber fractures and changes in the metal layers in the course of shear stress. Therefore, it can be concluded that the load is transmitted from one material to another through the adhesive layer in the overlap area where the resin is used as a carrier transmitting the loads.

## 4. Conclusions

The main idea of this article was to show the mechanism of FML failures after SLJ tests. The fractographic analysis of laminates based on CFRP/aluminum and GFRP/aluminum was demonstrated in the SEM observations. The principles of surface pretreatment were based on CAA and SAA. The surface morphology, surface roughness, SFE, and wetting angle analysis were considered as decisive factors of breaking shear stresses. In summary, the condition of the metal sheets after completed strength and morphological tests confirm the excellent bonding of individual components (i.e., between the metal and polymer composite). The presence of composite material (as well as fiber fragments) was detected on metal surfaces. It is evidence of the high adhesive and strength properties of the joint in metal–polymer composite systems and evidence of the correctly selected and completed process of metal surface preparation. Adhesion forces on the boundary of the anodized layer-composite were higher than the cohesion forces in the fiber composite. Both anodizing methods ensure an adhesive joint sustaining shear stress higher than 3 MPa, but the nature of the matrix cracking on the metal-composite boundary indicates differences depending on the method of anodizing applied. In both cases, the shear strength of the joint was higher for the layer configurations of ±45°. It can be concluded that the damage process in composite materials is mainly associated with fiber configuration in composite layers.

## Figures and Tables

**Table 1:** The values of wetting angle and surface free energy (SFE) on the anodized layers of the aluminum alloy.

Surface of 2024-T3 Aluminum Alloy	Wetting Angle θ [°]		Surface Free Energy (SFE) (mJ/m^2^)
	θ [°] Water	θ [°] Diiodomethane	γ_s_
Without surface preparation	67.27 ± 3.4	39.57 ± 2.4	43.9
CAA	16.45 ± 3.0	13.89 ± 3.23	72.2
SAA	27.1 ± 1.99	32.9 ± 2.99	66.4

**Table 2:** Surface roughness parameters for 2024-T3 aluminum after selected methods of surface preparation.

Type of Surface Preparation for 2024-T3 Aluminum Surface	Average Results For Surface Roughness Parameter R_a_ (µm)
No surface preparation	0.29 ± 0
CAA	0.29 ± 0.01
SAA	0.26 ± 0.05

**Table 3:** Results in the scope of strength properties for joints after shear tests.

Kind of FML	Type of Fibers	Anodizing Process	Configuration of Fibers Layers	Shear Strength (MPa)
Al/GFRP (0)_2_/Al	Glass	CAA+P	(0)	3.56 ± 0.35
Al/GFRP (±45)_2_/Al		CAA+P	(±45)	3.93 ± 0.54
Al/GFRP (0)_2_/Al		SAA+P	(0)	3.13 ± 0.05
Al/GFRP (±45)_2_/Al		SAA+P	(±45)	4.23 ± 0.21
Al/CFRP (0)_4_/Al	Carbon	CAA+P	(0)	4.14 ± 0.43
Al/CFRP (±45)_4_/Al		CAA+P	(±45)	5.69 ± 0.28
Al/CFRP (0)_4_/Al		SAA+P	(0)	3.92 ± 0.15
Al/CFRP (±45)_4_/Al		SAA+P	(±45)	3.85 ± 0.11

CAA+P = chromic acid anodizing + primer; SAA+P = sulfuric acid anodizing + primer.

**Table 4:** Dominated character of shear failure in the samples.

Kind of Sample	Character of Failure
Al/GFRP [0] CAA/Al	Adhesive—Cohesive (ADH+COH)
Al/GFRP [±45] CAA/Al	Adhesive—Cohesive (ADH+COH)
Al/GFRP [0] SAA/Al	Adhesive (ADH)
Al/GFRP [±45] SAA/Al	Adhesive (ADH)
Al/CFRP [0] CAA/Al	Adhesive—Cohesive (ADH+COH)
Al/CFRP [±45] CAA/Al	Adhesive—Cohesive (ADH+COH)
Al/CFRP [0] SAA/Al	Adhesive (ADH)
Al/CFRP [±45] SAA/Al	Adhesive—Cohesive (ADH+COH)
